# Community Resources and Hazards Across the Rural-Urban Continuum

**DOI:** 10.1001/jamanetworkopen.2026.4864

**Published:** 2026-04-03

**Authors:** Oshozimhede E. Iyalomhe, Shuo Jim Huang, Rozalina G. McCoy

**Affiliations:** 1 Department of Epidemiology and Human Genetics, University of Maryland School of Medicine, Baltimore; 2 University of Maryland Institute for Health Computing, North Bethesda; 3 Division of Endocrinology, Diabetes and Nutrition, Department of Medicine, University of Maryland School of Medicine, Baltimore

## Abstract

**Importance:**

Rural-urban differences in health outcomes have worsened over time, yet the variation in exposure to social determinants of health (SDOH), which are key drivers of these outcomes, has not been adequately quantified.

**Objective:**

To explore variation in exposure to a sample of beneficial and adverse community-level SDOH across the rural-urban continuum.

**Design, Setting, and Participants:**

This cross-sectional study included all residential addresses in Maryland using GPS-Health (Geographic Patterns of SDOH), a 2025 statewide address-level dataset.

**Main Outcomes and Measures:**

The straight-line (great-arc) distance to the nearest resource (hospital, federally qualified health center [FQHC], pharmacy, Supplemental Nutrition Assistance Program [SNAP] retailer, civic center, and major roadway) or hazard (gun violence incident, eviction site, and Environmental Protection Agency [EPA]–designated site) was estimated. Addresses were categorized using Rural-Urban Commuting Area codes as urban (1-3), large rural (4-6), small rural (7-9), and isolated rural (10). Linear mixed-effects models estimated adjusted differences by rurality.

**Results:**

The study included 2 070 970 addresses: 1 933 793 urban addresses (93.4%), 86 270 large-rural addresses (4.2%), 17 594 small-rural addresses (0.8%), and 33 313 isolated-rural addresses (1.6%). Adults aged 65 years and older were most prevalent in large-rural areas (30.8%), disability was more prevalent in small-rural areas (17.9%), and both were more common in isolated-rural than urban areas (older adults: 27.7% vs 17.1%; disability: 15.6% vs 11.6%). Compared with urban addresses in an adjusted model, isolated-rural addresses were farther from hospitals (estimated difference, 4.22 [95% CI, 3.32 to 5.13] miles), pharmacies (estimated difference, 2.16 [95% CI, 1.54 to 2.80] miles), SNAP retailers (estimated difference, 1.15 [95% CI, 0.83 to 1.47] miles), and civic centers (estimated difference, 1.09 [95% CI, 0.67 to 1.50] miles). FQHCs were closest to large-rural addresses (estimated difference, –2.13 [95% CI, –2.50 to –1.76] miles). Urban addresses were closest to hazards.

**Conclusions and Relevance:**

In this cross-sectional study of all residential addresses in Maryland, distance from health-promoting resources increased with rurality, with isolated-rural areas farthest, despite a higher representation of populations with greater health care needs. Our findings support examining rurality as a continuum rather than a dichotomy to better characterize health outcomes and SDOH variation and to inform place-specific interventions.

## Key Points

**Question:**

How does proximity to community resources and hazards differ by rurality?

**Findings:**

In this cross-sectional study of approximately 2.1 million Maryland addresses measuring straight-line distances, isolated rural areas—with high proportions of older adults and people with disabilities—were up to 5 miles farther from the nearest hospital, pharmacy, and Supplemental Nutrition Assistance Program retailer than urban addresses. Federally qualified health centers were closest to large-rural addresses.

**Meaning:**

The findings of this study suggest that using finer distinctions in categorizing rurality, rather than a simple urban-rural divide, could improve understanding of resource access and health impacts, informing place-tailored policies and interventions.

## Teaser

This cross-sectional study examines whether finer distinctions in the rural-urban continuum could help understand proximity to community-level resources, such as hospitals and pharmacies, and hazards, such as gun violence and eviction.

## Introduction

The differential distribution of resources and hazards that shape where people are born, live, work, and age—known as social determinants of health (SDOH)—is a major driver of health disparities.^1,2^ SDOH influence up to 70% of health outcomes but are unequally distributed across geography, including between rural and urban communities.^1,2,3,4,5^ These structural inequities have contributed to health disparities in the United States.^4,6,7^ For instance, rural residents face lower life expectancy (74.5 years in rural areas compared with 77.6 years in large metropolitan areas)^6^ and higher age-adjusted mortality,^4,6,7,8^ while incurring higher per capita health care costs despite lower health care utilization.^9,10^ These differences persist even after accounting for demographic factors including age, sex, and race.^8,11,12,13^

While the role of SDOH differences in driving health outcomes is well-established, the geographic patterns and domain-specific variations of SDOH remain poorly understood.^2^ Tools such as the Area Deprivation Index (ADI) and Social Vulnerability Index (SVI) use composite scores to quantify general patterns of deprivation, and prior studies have shown these indices capture similar underlying SDOH factors.^14^ However, by aggregating diverse socioeconomic and environmental domains into a single numeric score over large and often heterogeneous geographic units, such as counties, census tracts, or relatively homogenous block groups,^3^ these tools can obscure local variation, reduce accuracy in assessing individual-level risk, and offer limited actionable guidance compared with address-level information. Moreover, in heterogeneous areas where deprivation and resource richness coexist, vulnerable populations may be overlooked.^15^

Additionally, and most pertinent to rural health risks, area-level indices are limited in capturing how specific SDOH factors may vary.^2,16^ Rural communities may face limited health care access and geographic isolation, while urban communities may contend with greater environmental hazards, housing instability, and community safety concerns.^4,16^ Relying on dichotomous rural-urban categories—as is standard in most rural health–related studies—oversimplifies a continuum of place types,^17^ limiting their use in designing context-specific interventions.^4,15,18,19^

To address these limitations, prior work has pointed to the value of multidimensional spatial approaches in capturing complex SDOH patterns.^2^ Building on this, we examine and quantify how exposure to resources (SDOH that are hypothesized to be beneficial to health) and hazards (SDOH that are hypothesized to be harmful) varies across the rural-urban continuum in Maryland.^3^ We consider rurality as a continuum using the Rural-Urban Commuting Area (RUCA) classification, a census tract–level measure that is more spatially granular than county-based Rural-Urban Continuum Codes (RUCCs),^20,21^ grouping the 10-point RUCA into urban (RUCA 1-3; ≥50 000 people), large-rural (RUCA 4-6; 10 000-49 999 people), small-rural (RUCA 7-9; 2500–9999 people), and isolated-rural (RUCA 10; <2500 people) subgroups.^20,21^ We hypothesized that increasing rurality will be associated with progressively lower concentrations of both SDOH resources and hazards. By using high-resolution address-level data, we sought to capture local variation in SDOH exposure that is often missed in conventional area-level indices. We focus specifically on Maryland due to its geographic, demographic, and socioeconomic diversity, making it an illustrative example of why granular, nuanced examination of SDOH exposure is important.

## Methods

### Study Design and Data Sources

This cross-sectional study used a geospatial analytic infrastructure—Geographic Patterns of Social Determinants of Health (GPS-Health)—developed to quantify SDOH exposures at the address level.^3^ GPS-Health includes SDOH exposures from 10 domains that affect health: health care access, food access, economic environment, housing environment, educational access, transportation, civic access, community safety, gun violence, and environmental hazards. These domains were selected based on frameworks of kyriarchy (interconnected social systems), intersectionality (compounded disadvantage at sociodemographic intersections), and typologies of violence (systematic limitations on access to resources).^22,23^

GPS-Health was built using publicly available Maryland property parcel data compiled in January 2025.^3^ Briefly, geolocated residential addresses were identified from the Maryland Property Data Parcel Points Dataset and then linked to state and national datasets capturing SDOH features.^3^ GPS-Health incorporates data from across the United States to capture exposure to resources and hazards, even when relevant features extend beyond state boundaries.^3^ The University of Maryland Baltimore institutional review board determined that this study did not constitute human subjects research and was therefore exempt from review and the requirement for informed consent. This study followed the Strengthening the Reporting of Observational Studies in Epidemiology (STROBE) reporting guideline for cross-sectional studies.

### Study Population

The study included approximately 2.1 million Maryland residential addresses. These were designated as apartment, residential, town house, or mixed (residential-commercial).

### Outcome: Distance to Nearest SDOH Feature

The primary outcome was straight-line distance in miles from each address to the nearest SDOH feature (resource or hazard). Distances were calculated using QGIS version 3.34.2 and converted from degrees to miles using the Haversine formula.^24^ We multiplied angular distance (in degrees) by the constant π/180 × 3959.

SDOH domains align with the US Centers for Disease Control and Prevention’s (CDC) 5-domain SDOH framework: health care access and quality, education, neighborhood and built environment, social and community context, and economic stability; we excluded education because school catchment boundaries are broad and do not reflect address-level proximity.^25^ Resources were defined as features that promote access to health-supporting infrastructure or services. Health care access features included proximity to the nearest hospital, federally qualified health center (FQHC), or pharmacy; food access (neighborhood and built environment) was represented by Supplemental Nutrition Assistance Program (SNAP) retailers; social and community context was represented by civic centers and voting sites; and transportation infrastructure (neighborhood and built environment), by major roadways. Hazards were defined as features associated with health risk, including locations of recorded gun violence incidents (community safety) between 2015 and 2021, eviction sites (economic stability), and Environmental Protection Agency (EPA)–designated sites (neighborhood and built environment). Each address was linked to the nearest of each of the 9 SDOH features, yielding 9 distance measures per address, with an indicator variable specifying type of resource or hazard.

### Primary Exposure: Rural-Urban Status

Rural-urban status was the primary exposure, classified using RUCA codes, developed by the US Department of Agriculture’s Economic Research Service.^20^ RUCA codes categorize US census tracts based on population density, urbanization, and commuting patterns. We used the most current version available^20^ and grouped RUCA codes into 4 categories: urban (1-3), large rural (4-6), small rural (7-9), and isolated rural (10), consistent with prior public health research.^20,21^

### Covariates

We selected covariates based on prior literature.^4,11,16^ These included both address-level and census tract–level variables to capture economic and demographic contexts. At the address level, we obtained property value (in US dollars) from GPS-Health. Census tract–level covariates were obtained from the US Census Bureau’s 2023 American Community Survey via the Census Data API.^26^ These included median household income (in US dollars), race and ethnicity (self-reported), the percentage aged 65 years or older, and the percentage reporting a disability. GPS-Health assesses geographically distributed social drivers of risk, but it does not capture broader societal factors such as racism and discrimination that impact the geographic placement of resources and hazards. Therefore, it was important to consider census tract racial and ethnic composition as an additional factor in the model. Race and ethnicity were categorized as Hispanic, non-Hispanic Asian, non-Hispanic Black, non-Hispanic White, and non-Hispanic additional groups, which included non-Hispanic American Indian or Alaska Native, non-Hispanic Native Hawaiian or Other Pacific Islander, and non-Hispanic 2 or more races and/or some other race.

All census covariates were merged with residential addresses using census tract codes. We structured the data in long format, with each address having 1 row per resource or hazard. This resulted in 9 rows per address, 1 for each SDOH feature.

### Statistical Analysis

Continuous variables were summarized using means and SDs while categorical variables were described using frequencies and proportions. We excluded fewer than 2% of addresses due to missing covariates or a reported housing value of 0. Property values and household incomes were log-transformed. We examined the distribution of distances to resources and hazards by rural-urban status, visualized these distributions using density plots, and compared group means using analysis of variance.

To account for the hierarchical structure of the data (multiple distance measures per address, clustered within census tracts), we used linear mixed-effects models with random intercepts for census tracts, as the address-level random effect explained no variability. Additionally, prior studies have shown that the spatial arrangement of resources and hazards is not random and often varies by rurality.^27^ To reflect this, we assessed the heterogeneity in differences in distance to each resource or hazard type by rural-urban status. We tested this heterogeneity using a partial *F* test within the mixed-effects framework.

Covariates were included as confounders if they were supported by prior literature.^1,2,3,4,5^ We estimated adjusted differences in distance using a multivariable linear mixed-effects model, implemented in the lme4 R package fitting 3 models. Model 1 was stratified by type of resource or hazard with no additional covariate adjustment. Model 2 added the logarithm of property value and median household income and the percentage of residents who identified as White, were aged 65 years or older, or reported a disability. Model 3 was a fully adjusted model that included an interaction between rural-urban status and type of resource or hazard to allow rural-urban differences to vary by feature, and we present linear combinations relative to urban areas. All analysis was conducted using R version 4.3.3 (R Project for Statistical Computing). Statistical significance was defined at α = .05 using 2-sided tests, and 95% CIs are reported.

## Results

### Study Cohort

We identified 2 110 859 residential parcels in Maryland, which included addresses classified as residential, apartment, townhouse, or mixed residential-commercial. We then excluded 3629 addresses with missing data (eFigure 1 in Supplement 1), including 73 missing RUCA classification and 3556 missing tract-level median household income. An additional 36 260 addresses with a housing value of 0 were excluded. Addresses with missing data were more likely to be in urban or large-rural areas and had shorter distances to SDOH features (eTable 1 in Supplement 1). The final analytic sample included 2 070 970 residential addresses: 1 933 793 urban addresses (93.4%), 86 270 large-rural addresses (4.2%), 17 594 small-rural addresses (0.8%), and 33 313 isolated-rural addresses (1.6%).

Older adult and disability prevalence rates varied across the rural-urban continuum, with higher values observed in nonurban areas than in urban areas (Table 1). Adults aged 65 years and older were most prevalent in large-rural areas (30.8%), disability was more prevalent in small-rural areas (17.9%), and both were more common in isolated-rural than urban areas (older adults: 27.7% vs 17.1%; disability: 15.6% vs 11.6%). Lower-valued properties (<$150 000) were most prevalent in small-rural (46.9%) and isolated-rural (47.8%) areas, compared with urban (16.4%). Median incomes in small and isolated-rural areas were less than $110 000.

**Table 1.** Sociodemographic Characteristics and Straight-Line Distance to Resources and Hazards Across the Rural-Urban Continuum Among Residential Addresses in Maryland

Characteristic	Addresses, % (95% CI)^a^			
	Urban (n = 1 933 793)	Large rural (n = 86 270)	Small rural (n = 17 594)	Isolated rural (n = 33 313)
Demographic^b^				
Aged ≥65 y	17.07 (17.06-17.08)	30.84 (30.77-30.90)	22.26 (22.17-22.35)	27.70 (27.63-27.77)
Disabled	11.56 (11.55-11.57)	15.15 (15.13-15.16)	17.93 (17.86-18.00)	15.57 (15.53-15.61)
Race and ethnicity^b^				
Hispanic	10.38 (10.37-10.40)	5.86 (5.82-5.90)	4.53 (4.48-4.58)	2.76 (2.73-2.79)
Non-Hispanic Asian	6.18 (6.17-6.19)	1.34 (1.33-1.35)	1.03 (1.02-1.05)	0.63 (0.62-0.64)
Non-Hispanic Black	27.71 (27.67-27.75)	8.56 (8.48-8.64)	17.76 (17.57-17.96)	3.79 (3.74-3.84)
Non-Hispanic additional groups^c^	5.00 (5-5.01)	4.24 (4.22-4.25)	4.09 (4.06-4.12)	2.68 (2.66-2.7)
Non-Hispanic White	50.72 (50.68-50.76)	80.00 (79.90-80.10)	72.59 (72.37-72.80)	90.13 (90.06-90.20)
Housing value, $^d^				
<150 000	16.36 (16.31-16.42)	23.80 (23.51-24.08)	46.91 (46.18-47.65)	47.77 (47.23-48.30)
150 000 to <250 000	18.25 (18.19-18.30)	25.25 (24.96-25.54)	25.33 (24.69-25.98)	17.68 (17.27-18.09)
250 000 to <400 000	30.60 (30.53-30.66)	28.65 (28.34-28.95)	16.60 (16.05-17.15)	13.30 (12.94-13.67)
400 000 to <600 000	20.71 (20.66-20.77)	12.50 (12.28-12.72)	6.19 (5.83-6.55)	8.28 (7.99-8.58)
600 000 to <1 000 000	10.21 (10.16-10.25)	5.65 (5.49-5.8)	3.47 (3.2-3.74)	7.64 (7.36-7.93)
≥1 000 000	3.87 (3.85-3.9)	4.16 (4.03-4.3)	1.49 (1.32-1.67)	5.33 (5.08-5.57)
Median income, $^b^				
<81 000	23.38 (23.32-23.44)	49.41 (49.08-49.75)	59.77 (59.05-60.49)	42.79 (42.26-43.32)
81 000 to <110 000	24.02 (23.95-24.08)	38.47 (38.15-38.8)	40.23 (39.51-40.95)	57.21 (56.68-57.74)
110 000 to <145 000	26.81 (26.75-26.88)	12.11 (11.89-12.33)	0	0
≥145 000	25.79 (25.73-25.85)	0	0	0
Distance, mean (95% CI), miles				
Hospital	4.47 (4.46-4.47)	8.00 (7.97-8.04)	7.99 (7.9-8.08)	10.01 (9.96-10.06)
FQHC	4.99 (4.98-5.00)	6.99 (6.96-7.02)	5.13 (5.04-5.21)	5.45 (5.41-5.49)
Pharmacy	2.00 (2.00-2.00)	5.82 (5.79-5.85)	3.37 (3.33-3.42)	4.35 (4.32-4.38)
SNAP retailer	0.91 (0.91-0.91)	1.25 (1.24-1.26)	1.53 (1.51-1.56)	2.59 (2.57-2.61)
Civic center	1.12 (1.12-1.12)	2.26 (2.25-2.27)	2.29 (2.26-2.32)	4.02 (3.99-4.05)
Major roadway	3.67 (3.66-3.68)	26.93 (26.89-26.97)	17.43 (17.34-17.52)	12.85 (12.79-12.92)
EPA site	1.28 (1.28-1.28)	2.41 (2.40-2.43)	1.45 (1.43-1.47)	3.83 (3.81-3.86)
Eviction	1.17 (1.16-1.17)	1.98 (1.97-2.00)	2.91 (2.86-2.96)	5.30 (5.27-5.33)
Gun violence	1.23 (1.23-1.23)	3.05 (3.03-3.07)	3.63 (3.57-3.69)	6.97 (6.93-7.01)

Abbreviations: EPA, Environmental Protection Agency; FQHC, federally qualified health center; SNAP, Supplemental Nutrition Assistance Program.

^a^ Addresses classified by Rural-Urban Commuting Area groups: urban, 1 to 3; large rural, 4 to 6; small rural, 7 to 9; and isolated rural, 10. Data source was GPS-Health (Geographic Patterns of Social Determinants of Health).^3^

^b^ Assigned at the census tract level using 2023 American Community Survey data.

^c^ Includes non-Hispanic American Indian or Alaska Native, non-Hispanic Native Hawaiian or Other Pacific Islander, and non-Hispanic 2 or more and/or some other race.

^d^ Top coded at $10 million. Parcels above this threshold are included.

### Distance to Resources and Hazards by Rural-Urban Status

**Figure 1.**
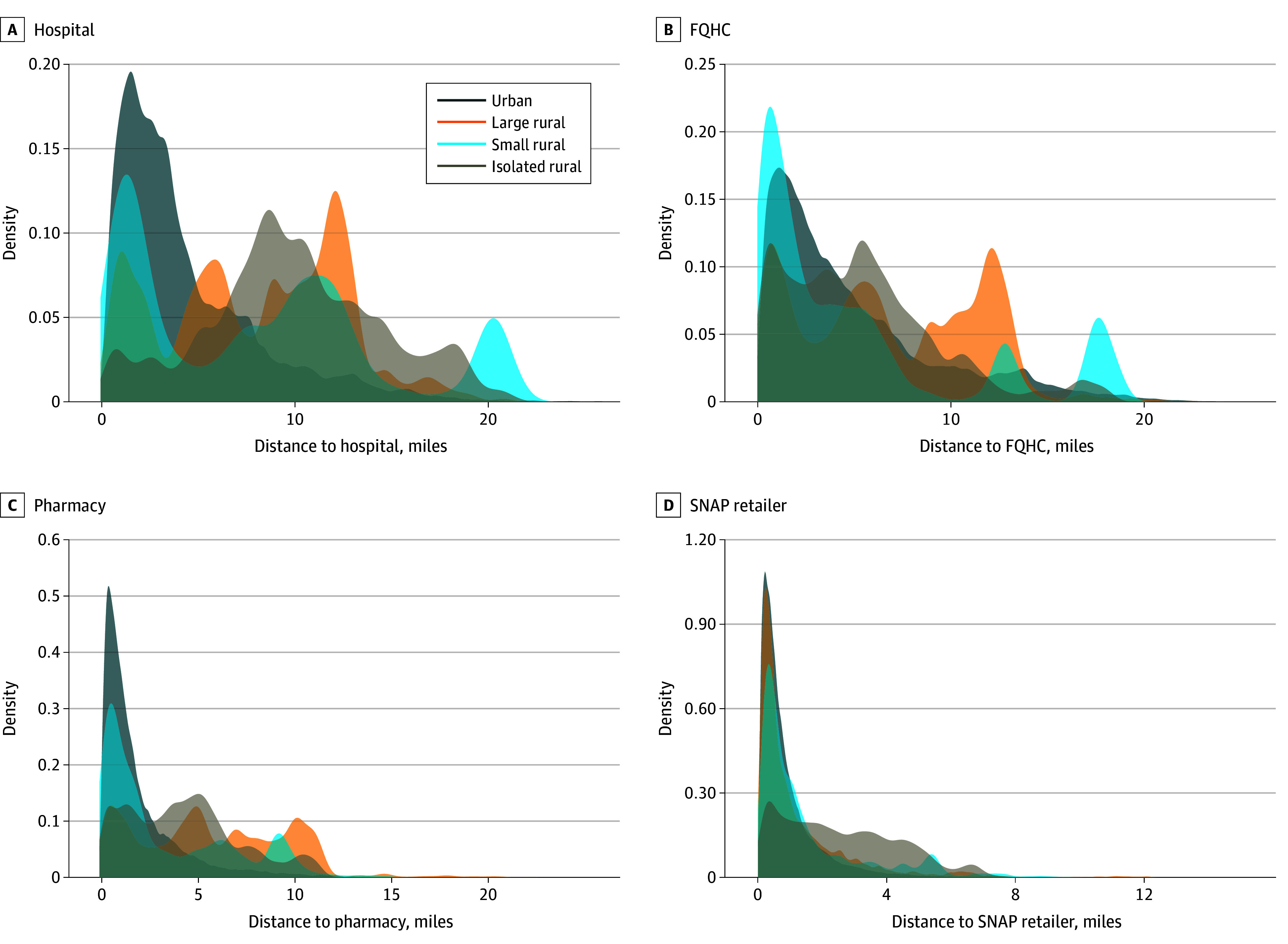
Density Distribution Graphs of Straight-Line Distance to Health Care and Food Resources Across the Rural-Urban Continuum Rural-urban categories classified according to the Rural-Urban Commuting Area codes: urban, 1 to 3; large rural, 4 to 6; small rural, 7 to 9; and isolated rural, 10. Data Source was GPS-Health (Geographic Patterns of Social Determinants of Health) publicly available Maryland property parcel data compiled in January 2025.^3^ FQHC indicates federally qualified health center; SNAP, Supplemental Nutrition Assistance Program.

**Figure 2.**
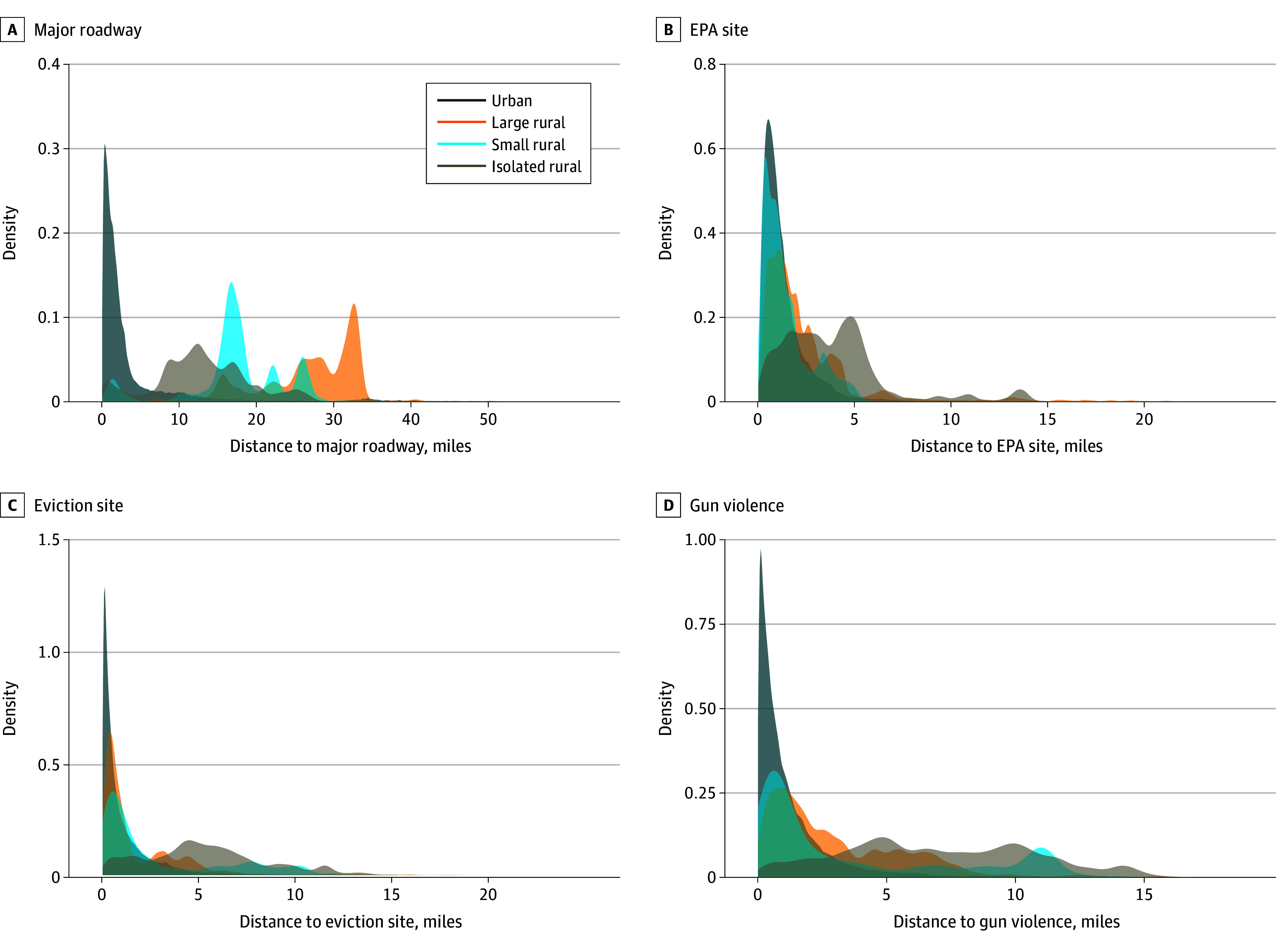
Density Distribution Graphs of Straight-Line Distance to Major Roadways and Hazards Rural-urban categories classified according to the Rural-Urban Commuting Area codes: urban, 1 to 3; large rural, 4 to 6; small rural, 7 to 9; and isolated rural, 10. Data Source was GPS-Health (Geographic Patterns of Social Determinants of Health) publicly available Maryland property parcel data compiled in January 2025.^3^ EPA indicates Environmental Protection Agency.

For most SDOH features, isolated-rural addresses were farthest. Distance to the nearest hospital was a mean 10.01 (95% CI, 9.96-10.06) miles in isolated-rural areas compared with 4.47 (95% CI, 4.46-4.47) miles in urban areas. Similar patterns were observed for civic centers (4.02 [95% CI, 3.99-4.05] vs 1.12 [95% CI, 1.12-1.12] miles) and SNAP retailers (2.59 [95% CI, 2.57-2.61] vs 0.91 [95% CI, 0.91-0.91] miles). Distances to hazards including EPA sites, eviction sites, and gun violence locations were also greatest in isolated-rural areas: with means of 3.83 (95% CI, 2.81-3.86), 5.30 (95% CI, 5.27-5.33), and 6.97 (95% CI, 6.93-7.01) miles, respectively. However, major roadways were farthest from large-rural areas (mean, 26.93 [95% CI, 26.89-26.97] miles). The shape of distance distribution varied by rural-urban status, becoming more dispersed with increasing rurality (Figures 1-2; eFigure 2 in Supplement 1). In isolated-rural areas, peaks (ie, the mode) occurred farther from both resources and hazards.

**Figure 3.**
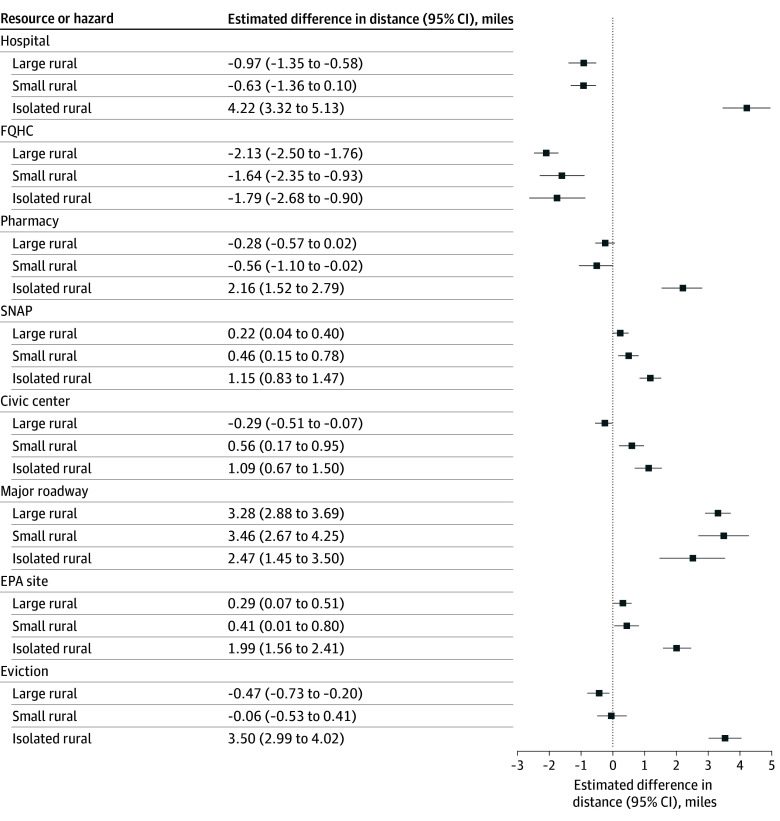
Dot Plot of Adjusted Differences in Straight-Line Distance Across the Rural-Urban Continuum by Resource or Hazard, Maryland Rural-urban categories classified according to the Rural-Urban Commuting Area codes: urban, 1 to 3; large rural, 4 to 6; small rural, 7 to 9; and isolated rural, 10. Model was a linear mixed-effects model stratified by resource or hazard, with a random intercept for census tract and adjusted for housing value and tract-level variables from the 2023 American Community Survey (percentage aged ≥65 years, percentage non-Hispanic White [self-reported], percentage with disability, and median household income). Data Source was GPS-Health (Geographic Patterns of Social Determinants of Health) publicly available Maryland property parcel data compiled in January 2025.^3^ EPA indicates Environmental Protection Agency; FQHC, Federally Qualified Health Center; SNAP, Supplemental Nutrition Assistance Program.

In unadjusted models stratified by type of resource or hazard (model 1), isolated-rural areas were farthest from the nearest feature across most domains compared with urban areas (eTable 2 in Supplement 1). After adjusting for sociodemographic and economic factors in stratified models (model 2), patterns remained consistent (Figure 3). Compared with urban addresses, isolated-rural addresses were farther from hospitals (estimated difference, 4.22 [95% CI, 3.32 to 5.13] miles), pharmacies (estimated difference, 2.16 [95% CI, 1.54 to 2.80] miles), SNAP retailers (estimated difference, 1.15 [95% CI, 0.83 to 1.47] miles), and civic centers (estimated difference, 1.09 [95% CI, 0.67 to 1.50] miles). FQHCs were closest to large-rural addresses (estimated difference, –2.13 [95% CI, –2.50 to –1.76] miles).

In the final model (model 3), including an interaction between rural-urban status and type of resource or hazard (Table 2), distance differences by rural-urban status statistically significantly varied by type of resource and hazard. Compared with urban areas, isolated-rural areas were farthest from hospitals (4.34 [95% CI, 3.61 to 5.07] miles), pharmacies (1.15 [95% CI, 0.42 to 1.88] miles), and civic centers (1.70 [95% CI, 0.97 to 2.43] miles). Compared with urban areas, FQHCs were closest in large-rural (–1.52 [95% CI, –1.88 to –1.17] miles) and small-rural (–1.52 [95% CI: –2.17 to –0.87] miles). Large-rural areas were farthest from major roadways (19.74 [95% CI, 19.38 to 20.10] miles). In the final model including census tract clustering, the intraclass correlation coefficient was 0.26.

**Table 2.** Adjusted Marginal Differences in Straight-Line Distance by Rural-Urban Commuting Area Group and SDOH Feature, Maryland, 2025^a^

SDOH feature	Urban (n = 1 933 793)	Estimate (95% CI)		
		Large rural (n = 86 270)	Small rural (n = 17 594)	Isolated rural (n = 33 313)
Hospital	0 [Reference]	0.02 (−0.34 to 0.37)	1.87 (1.22 to 2.52)	4.34 (3.61 to 5.07)
FQHC	0 [Reference]	−1.52 (−1.88 to −1.17)	−1.52 (−2.17 to −0.87)	−0.74 (−1.47 to −0.02)
Pharmacy	0 [Reference]	0.30 (−0.06 to 0.65)	−0.28 (−0.93 to 0.36)	1.15 (0.42 to 1.88)
SNAP	0 [Reference]	−3.18 (−3.53 to −2.82)	−1.03 (−1.68 to −0.38)	0.48 (−0.25 to 1.20)
Civic center	0 [Reference]	−2.38 (−2.74 to −2.02)	−0.49 (−1.13 to 0.16)	1.70 (0.97 to 2.43)
Major roadway	0 [Reference]	19.74 (19.38 to 20.10)	12.10 (11.46 to 12.75)	7.98 (7.25 to 8.71)
EPA site	0 [Reference]	−2.39 (−2.75 to −2.03)	−1.49 (−2.14 to −0.84)	1.35 (0.62 to 2.08)
Eviction	0 [Reference]	−2.71 (−3.06 to −2.35)	0.08 (−0.56 to 0.73)	2.93 (2.20 to 3.66)
Gun violence	0 [Reference]	−1.70 (−2.06 to −1.35)	0.74 (0.10 to 1.39)	4.53 (3.80 to 5.26)

Abbreviations: EPA, Environmental Protection Agency; FQHC, Federally Qualified Health Center; SDOH, social determinant of health; SNAP, Supplemental Nutrition Assistance Program.

^a^ Estimates are from model 3, a fully adjusted linear mixed-effects model with an interaction between Rural-Urban Commuting Area group and SDOH feature type, with a random intercept for census tract. The model adjusts for housing value and tract-level covariates from the 2023 American Community Survey: percentage aged 65 years and older, percentage Non-Hispanic White (self-reported), percentage with disability, and median household income. Reported estimates are the linear combination of main and interaction terms, comparing each group to the urban reference. Data source was GPS-Health publicly available Maryland property parcel data compiled in January 2025.^3^ Addresses classified by Rural-Urban Commuting Area groups: urban, 1 to 3; large rural, 4 to 6; small rural, 7 to 9; and isolated rural, 10.

## Discussion

Rural-urban differences in exposure to SDOH are independent drivers of health disparities even after accounting for demographics.^11^ However, detailed geographic patterns of these exposures have not been quantified.^2^ In this study, we measured address-level proximity to a range of health-related resources and hazards. In contrast to prior studies that have examined rurality as a dichotomous feature, often with inconsistent categorization of transition, fringe, or suburban areas as being rural or urban, we categorized rurality more granularly into 4 groups: urban, large rural, small rural, and isolated rural. This nuance proved to be important, as the 4 areas showed modest differences in straight-line distance to beneficial and adverse SDOH. Consistent with our hypothesis, distances to resources across addresses in Maryland generally increased with rurality, supporting prior work in rural health services research on the rural-urban continuum.^2,4,16^

Our work builds on and extends prior research on rural-urban differences in SDOH in several ways.^2,4,15,16,28^ We used a novel geospatial infrastructure, GPS-Health, which includes all residential addresses in Maryland and supports quantifying address-level proximity to SDOH features.^3^ This provides more granular geographic resolution than studies that rely on census tract data or limited address samples and better accounts for variation between individual addresses.^2,15,16,28^ By measuring proximity to individual resources and hazards, we identify detailed differences in exposure and distinct deprivation patterns.^16^ For example, while distance to most resources increased with rurality, this trend did not hold for FQHCs. Additionally, we captured the full geographic diversity of Maryland, including rural areas that are typically underrepresented in SDOH research.^16^ Our 4-level rurality classification supports prior work conceptualizing rurality as a continuum and extends this literature by quantifying how proximity to both resources and hazards varies across the continuum using address-level information.^2,4,16^ While this approach improves on binary classifications, heterogeneity likely remains within the 4 used rurality categories and warrants further investigation.

We found that binary rural-urban categories do not adequately capture variation in SDOH exposure using density distributions and analyses adjusted for demographic and economic factors. This aligns with prior work using clustering methods on more than 70 000 US census tracts to identify 7 distinct neighborhood typologies, illustrating a more complex pattern of deprivation.^2^ Our findings likely reflect the fact that many communities do not fit neatly into a rural-urban dichotomy, instead occupying positions along a continuum that combines mixed land use, overlapping vulnerabilities, and distinct coping strategies.^29^ Reliance on a dichotomy may obscure understanding of increasing rural-urban health disparities. More detailed rurality stratification in future research could clarify these patterns and support place-specific interventions.

In line with our hypothesis, our findings demonstrate that distance from resources and hazards generally increased with rurality. This aligns with research showing that rural US residents, on average, live twice as far from hospitals compared with urban or suburban residents.^28^ This pattern may reflect a reinforcing relationship between population density and resource distribution, whereby higher-density areas attract services and infrastructure, and greater resource availability in turn sustains population growth.^30^ Over time, this feedback may contribute to higher housing values, increased infrastructure investment, and service availability in urban-like areas, while lower population density in rural communities may limit demand, contribute to service closures, and reduce local capacity to cope with social and environmental stressors. Recent evidence documenting widespread rural hospital closures underscores how these dynamics may exacerbate existing access gaps and contribute to increasing health disparities.^31^

While greater distance from services in rural areas is well documented, to our knowledge, the present study is one of the first to quantify this gap at the address level across an entire state.^32^ This approach provides an essential basis for informing place-based policies and supporting efficient resource allocation, including addressing concentrated hazards in urban-like areas and resource remoteness in rural-like areas. Importantly, adding facilities indiscriminately may not address this challenge, as a study in Wisconsin found that while proximity to health care facilities improved utilization, the added benefit diminished after the first 1 or 2 additional sites.^32^

Finally, we found that isolated-rural areas combined remoteness from resources with relatively higher representation of older adults and individuals with disabilities as well as those with lower median incomes. Our findings reflect known national trends: rural populations are older, have more chronic health conditions and mobility impairments, and face greater financial insecurity.^4,6,33^ These factors reflect a greater need for health and supportive resources. These combined factors signal greater health care needs. However, the compounded burden of age, disability, limited financial means, and greater distances from care can reduce service use, delay treatment, increase complications, and lead to more preventable hospitalizations and higher care costs.^9,10^

### Limitations and Strengths

Our findings should be interpreted considering several study limitations. First, we used straight-line distance to estimate remoteness, which does not account for road networks or traffic conditions.^34^ While this may have less impact when assessing proximity to hazards, it is more relevant for access to services.^34^ However, straight-line distance is a practical and reasonable proxy for travel distance in policy planning.^34^ Prior studies have found a strong correlation between straight-line and travel distances, with only local exceptions near physical barriers such as shorelines.^34^ Also, because straight-line distance typically underestimates actual travel, our results may represent a conservative estimate of remoteness.^34^ Second, travel time may offer a more meaningful measure than distance.^35^ However, literature suggests that both measures are important.^35^ Travel time tends to have less influence over shorter distances and becomes more significant as distance increases.^35^ Finally, the RUCA classifications used in our analysis reflect the most current publicly available data but do not capture the most recent population or development shifts.

Nevertheless, our study has important strengths. First, the GPS-Health dataset combines address-level information from various Maryland state agencies, and out-of-state sources to better capture exposures near the Maryland border.^3^ This allowed us to examine, in a novel way, individual-level exposures to the extent previously infeasible. Second, while this was a single state analysis, Maryland may be considered a snapshot of the United States, with a diverse mix of urban and rural areas, and substantial socioeconomic heterogeneity, despite its otherwise small landmass.^3^ This is important given that rural populations are frequently underrepresented in research.^16^ Finally, rather than collapsing different SDOH domains into a single summary score, we analyzed them separately to better capture how specific resources vary across the rural–urban continuum. This approach is supported by increasing evidence that examining spatial and contextual factors individually provides a more accurate understanding of the conditions that shape health.^2^

## Conclusions

In this cross-sectional study of all residential addresses across Maryland, we found that distance from resources increased with rurality and that isolated-rural areas were farthest from health-promoting resources, despite having a high representation of populations with greater health care needs, including older adults and individuals with disabilities. Our findings support future research using more detailed rurality along the rural-urban continuum to better understand increasing health and geographic disparities. Importantly, expanding the GPS-Health infrastructure to include additional domains, network-based travel distances, and travel time could further improve our understanding of spatial differences in exposure to resources and hazards. GPS-Health also offers value to researchers working with electronic health records, claims, and other health-related data by helping account for neighborhood-level SDOH differences that influence health care use and health outcomes. These insights have important implications for public health researchers, clinicians, hospital administrators, policymakers, and others focused on improving equitable access to health care and related services.

## Notes

Supplement 1. **eFigure 1.** Flow Diagram of Residential Parcel Inclusion and Exclusion Criteria Using GPS-Health (Geographic Patterns of Social Determinants of Health), Maryland, 2025 **eTable 1.** Comparison of Residential Addresses Included in the Analytic Sample vs Excluded Due to Missing Data **eFigure 2.** Density Distribution of Straight-Line Distance to Resources and Hazards Across the Rural-Urban Continuum **eTable 2.** Unadjusted Differences in Straight-Line Distance Across the Rural-Urban Continuum by Resource or Hazard, Maryland

Supplement 2.
Data Sharing Statement
