# Supplementary material for: Community Resources and Hazards Across the Rural-Urban Continuum
Source: JAMA Netw Open. 2026 Apr 3;9(4):e264864. doi: 10.1001/jamanetworkopen.2026.4864 (PMC13049494; doi:10.1001/jamanetworkopen.2026.4864)
Supplement: Supplement 1. — eFigure 1. Flow Diagram of Residential Parcel Inclusion and Exclusion Criteria Using GPS-Health (Geographic Patterns of Social Determinants of Health), Maryland, 2025 eTable 1. Comparison of Residential Addresses Included in the Analytic Sample vs Excluded Due to Missing Data eFigure 2. Density Distribution of Straight-Line Distance to Resources and Hazards Across the Rural-Urban Continuum eTable 2. Unadjusted Differences in Straight-Line Distance Across the Rural-Urban Continuum by Resource or Hazard, Maryland [file jamanetwopen-e264864-s001.pdf]

Supplemental Online Content

Iyalomhe OE, Huang SJ, McCoy RG. Community resources and hazards across the rural-urban continuum. *JAMA Netw Open.* 2026;9(4):e264864. doi:10.1001/jamanetworkopen.2026.4864

eFigure 1. Flow Diagram of Residential Parcel Inclusion and Exclusion Criteria Using GPS-Health (Geographic Patterns of Social Determinants of Health), Maryland, 2025

eTable 1. Comparison of Residential Addresses Included in the Analytic Sample vs Excluded Due to Missing Data

eFigure 2. Density Distribution of Straight-Line Distance to Resources and Hazards Across the Rural-Urban Continuum

eTable 2. Unadjusted Differences in Straight-Line Distance Across the Rural-Urban Continuum by Resource or Hazard, Maryland

This supplemental material has been provided by the authors to give readers additional information about their work.

© 2026 Iyalomhe OE et al. *JAMA Network Open*.

eFigure 1. Flow Diagram of Residential Parcel Inclusion and Exclusion Criteria Using GPS-Health (Geographic Patterns of Social Determinants of Health), Maryland, 2025

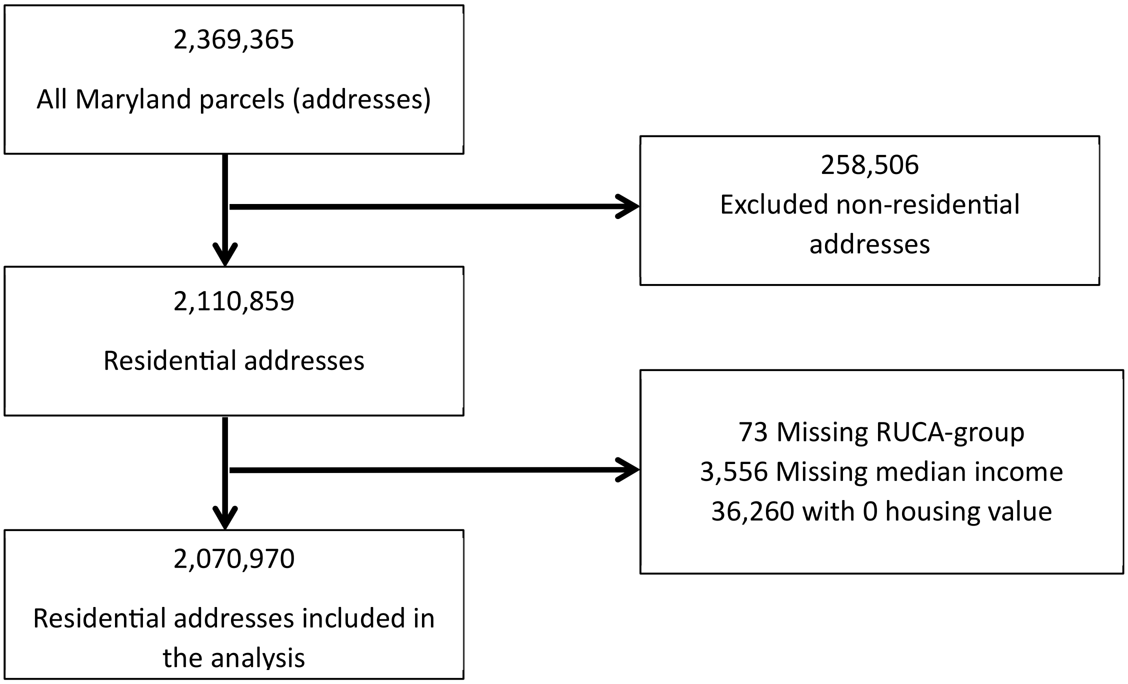

© 2026 Iyalomhe OE et al. *JAMA Network Open*.

eTable 1. Comparison of Residential Addresses Included in the Analytic Sample vs Excluded Due to Missing Data

SDOH Feature (N)	Complete	Missing ≥1 variable
	2,070,970	39,889
**RUCA group (%)**		
Urban	93.4	97.6
Large Rural	4.2	1.4
Small Rural	0.8	0.2
Isolated Rural	1.6	0.8
**Demographics**[a]		
Aged 65+ (%)	17.86	16.49
Disabled (%)	11.83	11.23
Non-Hispanic White (%)	52.76	44.19
Non-Hispanic Black (%)	26.45	31.29
Hispanic (%)	10.02	9.97
Non-Hispanic Asian (%)	5.85	9.24
Non-Hispanic Other[c] (%)	4.93	5.3
**Mean Distance**		
Hospital (miles)	4.73	4.37
FQHC (miles)	5.08	4.84
Pharmacy (miles)	2.21	2.02
SNAP (miles)	0.95	0.92
CVC (miles)	1.22	1.09
Major Roadway (miles)	4.9	4.32
EPA Site (miles)	1.37	1.3
Eviction (miles)	1.28	1.12
Gun Violence (miles)	1.42	1.2

RUCA Group: based on US Department of Agriculture codes: Urban (1–3), Large Rural (4–6), Small Rural (7–9), Isolated Rural (10); EPA = Environmental Protection Agency; FQHC = Federally Qualified Health Center; SNAP = Supplemental Nutrition Assistance Program; [a] Assigned at the census tract level using 2023 American Community Survey data; [c] Other includes non-Hispanic American Indian or Alaska Native, non-Hispanic Native Hawaiian or Other Pacific Islander, and non-Hispanic Two or more and/or Some Other Races. Data Source[3]: GPS-Health (Geographic Patterns of Social Determinants of Health) (doi.org/10.5281/zenodo.14422743).

© 2026 Iyalomhe OE et al. *JAMA Network Open*.

eFigure 2. Density Distribution of Straight-Line Distance to Resources and Hazards Across the Rural-Urban Continuum

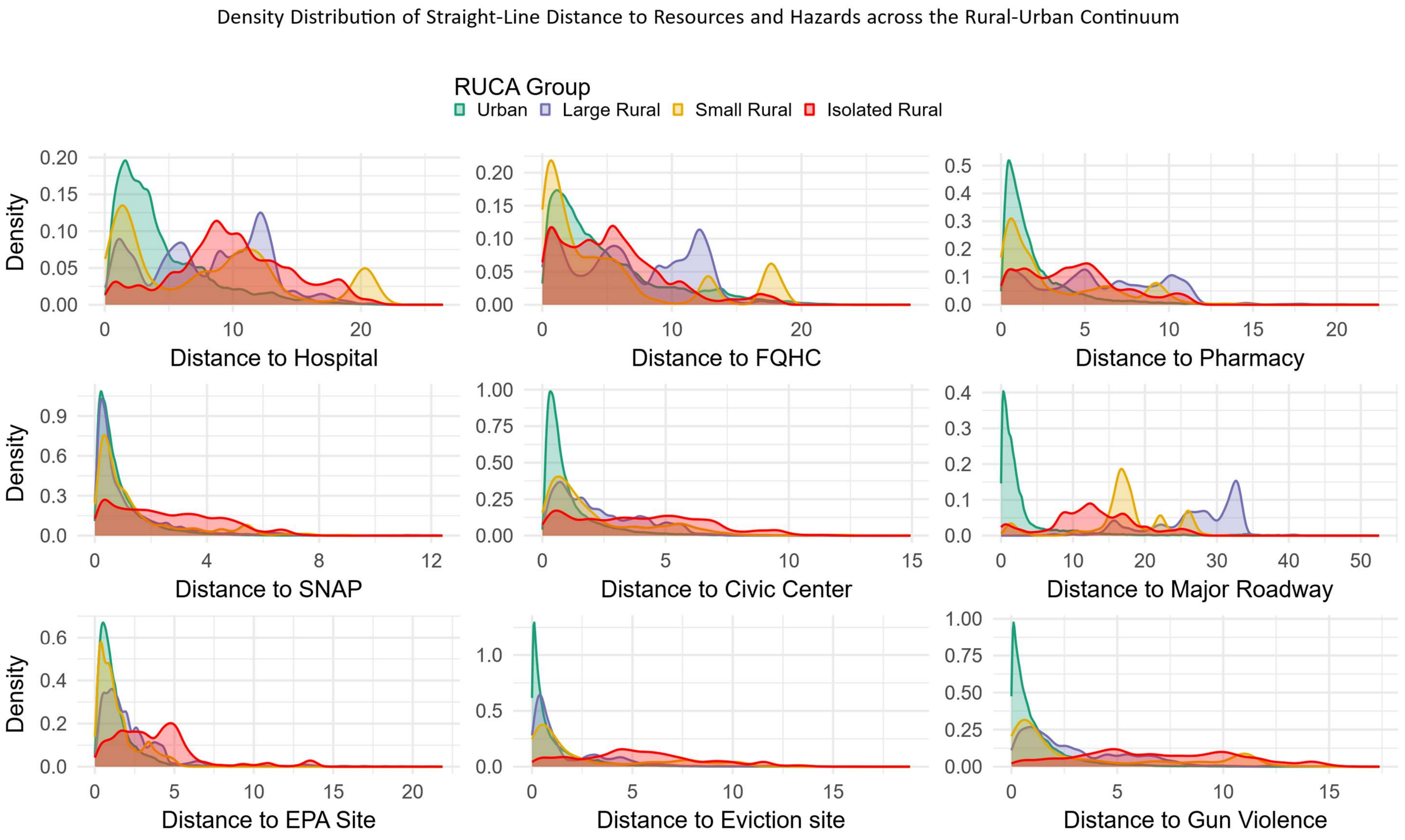

Distances increase with rurality, but patterns vary by feature.

RUCA Group: based on US Department of Agriculture codes: Urban (1–3), Large Rural (4–6), Small Rural (7–9), Isolated Rural (10);
FQHC = Federally Qualified Health Center; SNAP = Supplemental Nutrition Assistance Program; EPA = Environmental Protection Agency;
Data Source[3]: GPS-Health (Geographic Patterns of Social Determinants of Health): publicly available Maryland property parcel data compiled in January 2025

© 2026 Iyalomhe OE et al. *JAMA Network Open*.

eTable 2. Unadjusted Differences in Straight-Line Distance Across the Rural-Urban Continuum by Resource or Hazard, Maryland

SDOH Feature (N)	Urban (1,933,793)	Large Rural (86,270)		Small Rural (17,594)		Isolated Rural (33,313)	
		Estimate	95% CI	Estimate	95% CI	Estimate	95% CI
Hospital	Ref	-0.84	[-1.22, -0.45]	-0.33	[-1.07, 0.41]	4.70	[3.81, 5.65]
FQHC	Ref	-2.00	[-2.37, -1.62]	-1.32	[-2.05, -0.60]	-1.24	[-2.16, -0.31]
Pharmacy	Ref	-0.20	[-0.50, 0.09]	-0.39	[-0.94, 0.16]	2.53	[1.88, 3.18]
SNAP	Ref	0.28	[0.09, 0.46]	0.57	[0.23, 0.90]	1.37	[1.02, 1.72]
Civic Center	Ref	-0.15	[-0.38, 0.07]	0.82	[0.41, 1.22]	1.45	[1.01, 1.88]
Major Roadway	Ref	3.36	[2.95, 3.76]	3.66	[2.86, 4.45]	2.78	[1.75, 3.80]
EPA Site	Ref	0.33	[0.11, 0.55]	0.48	[0.08, 0.88]	2.14	[1.70, 2.57]
Eviction	Ref	-0.37	[-0.64, -0.10]	0.11	[-0.38, 0.59]	3.95	[3.40, 4.50]
Gun Violence	Ref	1.45	[1.19, 1.70]	1.95	[1.49, 2.41]	4.84	[4.31, 5.36]

RUCA Group: based on US Department of Agriculture codes: Urban (1–3), Large Rural (4–6), Small Rural (7–9), Isolated Rural (10);
95% CI: 95% Confidence Interval;
EPA = Environmental Protection Agency; FQHC = Federally Qualified Health Center; SNAP = Supplemental Nutrition Assistance Program;
Model 1: Linear mixed-effects models stratified by feature, with random intercept for census tract; no covariates included.
Data Source [3]: GPS-Health (Geographic Patterns of Social Determinants of Health) publicly available Maryland property parcel data compiled in January 2025.

© 2026 Iyalomhe OE et al. *JAMA Network Open*.
