# Supplementary material for: Community Resources and Hazards Across the Rural-Urban Continuum
Source: JAMA Netw Open. 2026 Apr 3;9(4):e264864. doi: 10.1001/jamanetworkopen.2026.4864 (PMC13049494; doi:10.1001/jamanetworkopen.2026.4864)
Supplement: Supplement 2. — Data Sharing Statement [file jamanetwopen-e264864-s002.pdf]

Data Sharing Statement

Iyalomhe. Community Resources and Hazards Across the Rural-Urban Continuum. *JAMA Netw Open*. Published April 03, 2026. doi:10.1001/jamanetworkopen.2026.4864

Data
Data available: Yes
Data types: Data (not involving human participants)
How to access data: GPS-Health data are available at https://doi.org/10.5281/zenodo.14422743
When available: With publication

Supporting Documents
Document types: None

Additional Information
Who can access the data: anyone requesting the data
Types of analyses: for any purpose
Mechanisms of data availability: with investigator support
